# Upper-Limb Compartment Syndrome Following Rapid Infusion Catheter Extravasation During Orthotopic Liver Transplantation: A Case Report

**DOI:** 10.7759/cureus.116959

**Published:** 2026-09-26

**Authors:** Laurence Weinberg, Haoran Liu, Jemin Suh, Daniel Laas, Nattaya Raykateeraroj

**Affiliations:** 1 Department of Anesthesiology, Austin Health, Melbourne, AUS; 2 Department of Anesthesiology, Austin Health, Heidelberg, AUS; 3 Department of Anesthesiology, Faculty of Medicine, Siriraj Hospital, Mahidol University, Bangkok, THA

**Keywords:** acute compartment syndrome, extravasation injury, fasciotomy, liver transplantation, massive transfusion, rapid infusion catheter, vascular access complication

## Abstract

Rapid infusion catheters (RICs) provide high-flow peripheral venous access for major haemorrhage but can cause severe tissue injury if pressurised infusion becomes extravascular. We report upper-limb acute compartment syndrome during orthotopic liver transplantation following transfusion through an 8.5 Fr Arrow RIC (Teleflex Incorporated, Morrisville, NC, USA). A female in her late 60s with decompensated cryptogenic cirrhosis underwent urgent transplantation. Four hours before surgery, an attempt to place an 8.5 Fr RIC in the left antecubital fossa was abandoned because of guidewire resistance. Immediately before incision, a second 8.5 Fr RIC was inserted at the same site under real-time ultrasound guidance; intraluminal position was confirmed and a 20 mL saline flush produced no resistance or swelling. Approximately 4.5 L of blood products and resuscitation fluid were subsequently delivered through the circuit over two hours, although the proportion that extravasated could not be determined. During the dissection phase, potassium increased to 6.1 mmol/L and lactate to 5.2 mmol/L. Examination beneath the drapes revealed a cool, dusky hand with an absent radial pulse, a tense anterior compartment of the upper arm, and ecchymosis, epidermolysis, and haemorrhagic bullae extending from the antecubital fossa into the proximal forearm. Infusion was stopped immediately, and surgical decompression of the anterior compartment of the upper arm was performed in parallel with the transplant. The radial pulse returned immediately after fascial release, and full motor and sensory function was present at discharge and at six-week follow-up. This case demonstrates that successful ultrasound confirmation and an unremarkable test flush at insertion do not ensure continued intravascular function during positioning, draping, and pressurised infusion. Persistent or unexplained guidewire resistance warrants reassessment, and a potentially traumatised vein should not be reused for high-pressure infusion when safer alternative access is available. High-flow peripheral access sites should remain visible whenever feasible or undergo a defined programme of reinspection throughout use.

## Introduction

Orthotopic liver transplantation is associated with abrupt haemodynamic instability and the potential for major haemorrhage, making reliable high-flow venous access an essential component of perioperative preparation [1,2]. Rapid infusion catheters (RICs) are large-bore peripheral sheaths placed using the Seldinger technique and, when coupled to a rapid infusion system, can achieve flow rates exceeding those of conventional peripheral cannulae and of many multi-lumen central venous catheters, although the flow achievable depends on catheter length, internal diameter, and lumen configuration, as well as on the infusion system used [3-5]. Their peripheral location avoids some complications of large-bore central venous access, but extravasation during pressurised infusion can produce rapid and extensive soft-tissue injury.

Acute compartment syndrome (ACS) occurs when pressure within a closed fascial compartment compromises tissue perfusion, with progressive ischaemia and irreversible neuromuscular injury if decompression is delayed [6]. Intravenous infiltration is an uncommon but recognised cause. In a systematic review of forearm ACS, intravenous infiltration accounted for seven of 84 cases (8.3%) [7]. In the largest published series of RIC use during liver transplantation, 14 of 839 recipients (1.67%) experienced a catheter-related complication and seven required surgical consultation; none required fasciotomy or wound debridement and no permanent sequelae were reported [8]. Severe RIC-related tissue injury has nevertheless been described [9], and compartment syndrome following pressurised peripheral infusion is well documented [10,11].

Recognition during general anaesthesia is particularly difficult. Pain and paraesthesia cannot be reported, motor examination is unavailable, and the affected limb may be obscured by surgical drapes. Liver-transplant guidance therefore recommends regular review of high-flow vascular access sites for extravasation and haematoma [2]. We report RIC extravasation during orthotopic liver transplantation that progressed to upper-limb ACS requiring urgent surgical decompression. The case illustrates the limitations of insertion-time verification and the importance of continued surveillance after final positioning and during pressurised infusion.

## Case presentation

A female in her late 60s with decompensated cryptogenic cirrhosis was admitted while awaiting orthotopic liver transplantation. She presented with worsening abdominal pain, abdominal distension, and peripheral oedema. Admission laboratory investigations demonstrated anaemia, thrombocytopenia, coagulopathy, hyperbilirubinaemia, and mild hyperlactataemia (Table 1). Her recorded Model for End-Stage Liver Disease score was 27. The patient provided written informed consent for publication of this report and the accompanying images.

**Table 1:** Selected laboratory findings on admission. eGFR, estimated glomerular filtration rate.

Laboratory test (units)	Value	Reference range
Haemoglobin (g/L)	80	115-155
Platelets (×10⁹/L)	81	150-400
Creatinine (µmol/L)	88	45-90
eGFR (mL/min/1.73 m²)	59	>90
Sodium (mmol/L)	139	135-145
Potassium (mmol/L)	4.2	3.5-5.2
International normalised ratio	3.0	0.8-1.2
Bilirubin (µmol/L)	162.8	<21.0
Lactate (mmol/L)	2.6	0.5-2.0

Over the subsequent two weeks, hepatic encephalopathy worsened and progressive oliguric renal failure developed despite more than five days of terlipressin therapy. Haemoglobin fell to 60 g/L because of upper gastrointestinal bleeding from portal hypertensive gastropathy. On the evening before transplantation, she was admitted to the intensive care unit for continuous renal replacement therapy in the setting of uraemia, encephalopathy, and an increasing oxygen requirement. Following multidisciplinary discussion among the intensive care, anaesthetic, and liver-transplant teams and the patient's family, transplantation proceeded in recognition of the high perioperative risk.

Because of ongoing anaemia and the anticipated risk of major intraoperative haemorrhage, large-bore venous access was prioritised. An 8.5 Fr Arrow® RIC (Teleflex Incorporated, Morrisville, NC, USA) was attempted under ultrasound guidance in the left antecubital fossa in the intensive care unit. Resistance was encountered during guidewire advancement, and the attempt was abandoned; the sheath was not inserted, and no fluid or medication was administered through the site. The failed attempt was documented but was not formally communicated to the operative team as a site to avoid for subsequent high-pressure access.

Four hours later, immediately before surgical incision, an 8.5 Fr RIC was inserted at the same left antecubital site by a consultant anaesthetist experienced in ultrasound-guided RIC placement, under real-time ultrasound guidance, at the first pass and without apparent difficulty. Before insertion, the site showed no bruising, swelling, or haematoma, and the vein appeared patent and compressible on ultrasound. Venous entry and intraluminal guidewire position were confirmed dynamically on ultrasound; however, no still image or video clip was archived because the procedure was undertaken as part of routine clinical care. The dilator was removed, and a 20 mL saline flush was administered without resistance, swelling, or leakage. The left upper limb was then abducted on an arm board and covered by warming and surgical drapes, preventing direct visual inspection during the procedure. Continuous renal replacement therapy was interrupted for transfer to theatre and was not continued intraoperatively.

Additional access and monitoring followed the institutional liver-transplant protocol. A second 8.5 Fr Arrow® RIC was inserted under ultrasound guidance in a non-flexion site on the right forearm. Central venous access comprised a right internal jugular 9 Fr Arrow® MAC introducer (Teleflex Incorporated) supporting a Swan-Ganz VIP pulmonary artery catheter (Edwards Lifesciences, Irvine, CA, USA), together with a separate multi-lumen central venous catheter for vasoactive infusions. In accordance with our standardised liver-transplant set-up, in which a femoral arterial catheter is inserted for every recipient, arterial monitoring comprised a right femoral arterial line, used for continuous blood pressure measurement, and a left radial arterial line, used predominantly for intermittent blood sampling because it is readily accessible to the anaesthetist at the arm board and its use for sampling avoids repeated interruption of the femoral pressure trace. No non-invasive blood pressure cuff was applied to the left arm, and no other infusions were administered through that limb. Transoesophageal echocardiography was used throughout the transplant.

Resuscitation through the left RIC was delivered with a Belmont RI-2 rapid infuser (Belmont Medical Technologies, Billerica, MA, USA). The line-pressure limit was 300 mmHg, the default setting on system start-up, and no high-pressure alarm was documented during use of the affected circuit. The infusion line was connected directly to the RIC hub. Fluids were administered intermittently as 200 mL boluses at a set rate of 200 mL/min in response to blood loss and haemodynamic requirements. During the approximately two hours between commencement of transfusion and recognition of the limb injury, four units of packed red blood cells, 1.1 L of autologous cell-salvaged blood, 2 L of Plasma-Lyte 148, and 400 mL of 20% albumin were delivered through this circuit, a cumulative volume of approximately 4.5 L. The proportion of this volume that remained intravascular versus extravasated could not be determined.

Approximately two hours after pressurised transfusion commenced, during the dissection phase of transplantation, arterial blood gas analysis of a sample drawn from the left radial arterial line showed a potassium concentration of 6.1 mmol/L and lactate of 5.2 mmol/L. By comparison, the sample obtained after induction of anaesthesia had shown a potassium level of 4.1 mmol/L and lactate level of 2.6 mmol/L, and the preceding intraoperative sample, obtained approximately 30 minutes earlier, had shown a potassium level of 4.6 mmol/L and lactate level of 2.7 mmol/L. Because the radial catheter lay distal to the concealed access site, the analysis was immediately repeated on a sample drawn from the right femoral arterial line; the results were identical, indicating a systemic abnormality rather than a local sampling artefact from a poorly perfused limb. The femoral line was used for all subsequent blood sampling. Both abnormalities have multiple potential causes during liver transplantation, and, in this patient, oliguric renal failure with interruption of continuous renal replacement therapy and substantial red-cell transfusion were each plausible contributors; nevertheless, their magnitude and timing were discordant with the operative stage and prompted systematic re-examination. Retrospective review of the anaesthetic record subsequently showed progressive damping of the left radial arterial waveform over the same interval. Because the right femoral line was the primary continuous pressure monitor and remained normal, this change was not recognised as a regional perfusion warning at the time.

**Figure 1:**
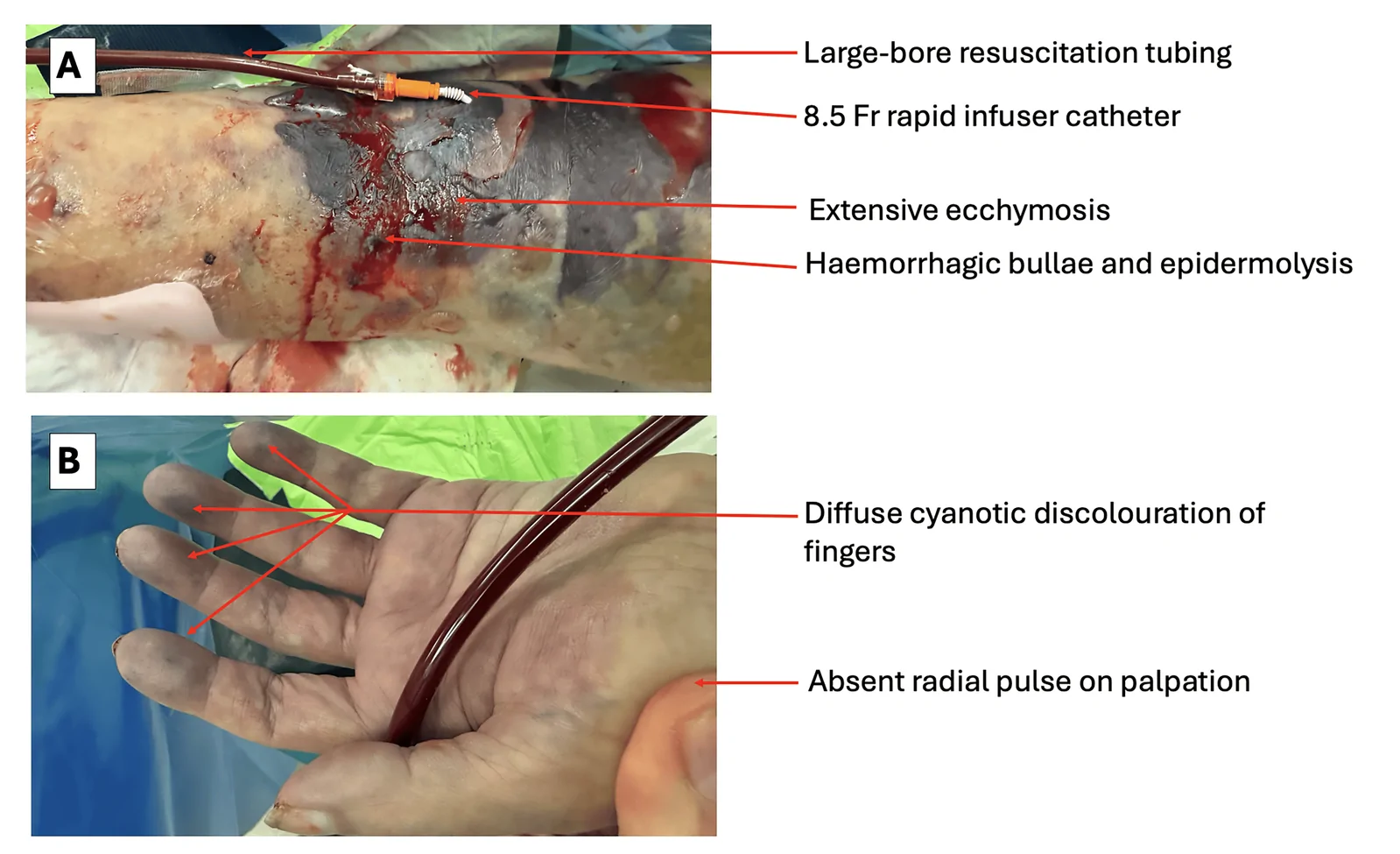
Clinical appearance at recognition of the complication. (A) Marked swelling and extensive ecchymosis at the left antecubital fossa and proximal volar forearm, with haemorrhagic bullae and epidermolysis adjacent to the rapid infusion catheter site. (B) Dusky discolouration of the digits with an absent radial pulse on palpation. The photographs are used with the patient's written informed consent.

Inspection beneath the drapes revealed a cool, dusky left hand with an absent radial pulse. The cutaneous changes, comprising extensive ecchymosis, epidermolysis, and haemorrhagic bullae, were centred on the antecubital fossa and extended distally into the proximal volar forearm (Figure 1). The tense swelling, in contrast, was centred on the distal upper arm, corresponding to the anterior compartment in which the catheter tip lay; the volar forearm, although ecchymotic, was less tense, and the dorsal forearm and hand were soft. Motor and sensory examination was not possible under general anaesthesia. The combination of a tense anterior upper-arm compartment, impaired distal perfusion, and established skin injury was considered sufficient clinical evidence of threatened limb perfusion to justify immediate surgical treatment; compartment-pressure measurement was not performed because it would not have altered the decision for urgent decompression. The plastic surgical team was called immediately.

Infusion through the left RIC was stopped immediately, and the catheter was left temporarily in situ to avoid additional manipulation while the limb was assessed. The right arm was fully inspected and remained soft and normally perfused, without swelling or discolouration at the contralateral RIC site. Subsequent volume resuscitation was delivered through the right-sided RIC, which functioned normally for the remainder of the transplant.

**Figure 2:**
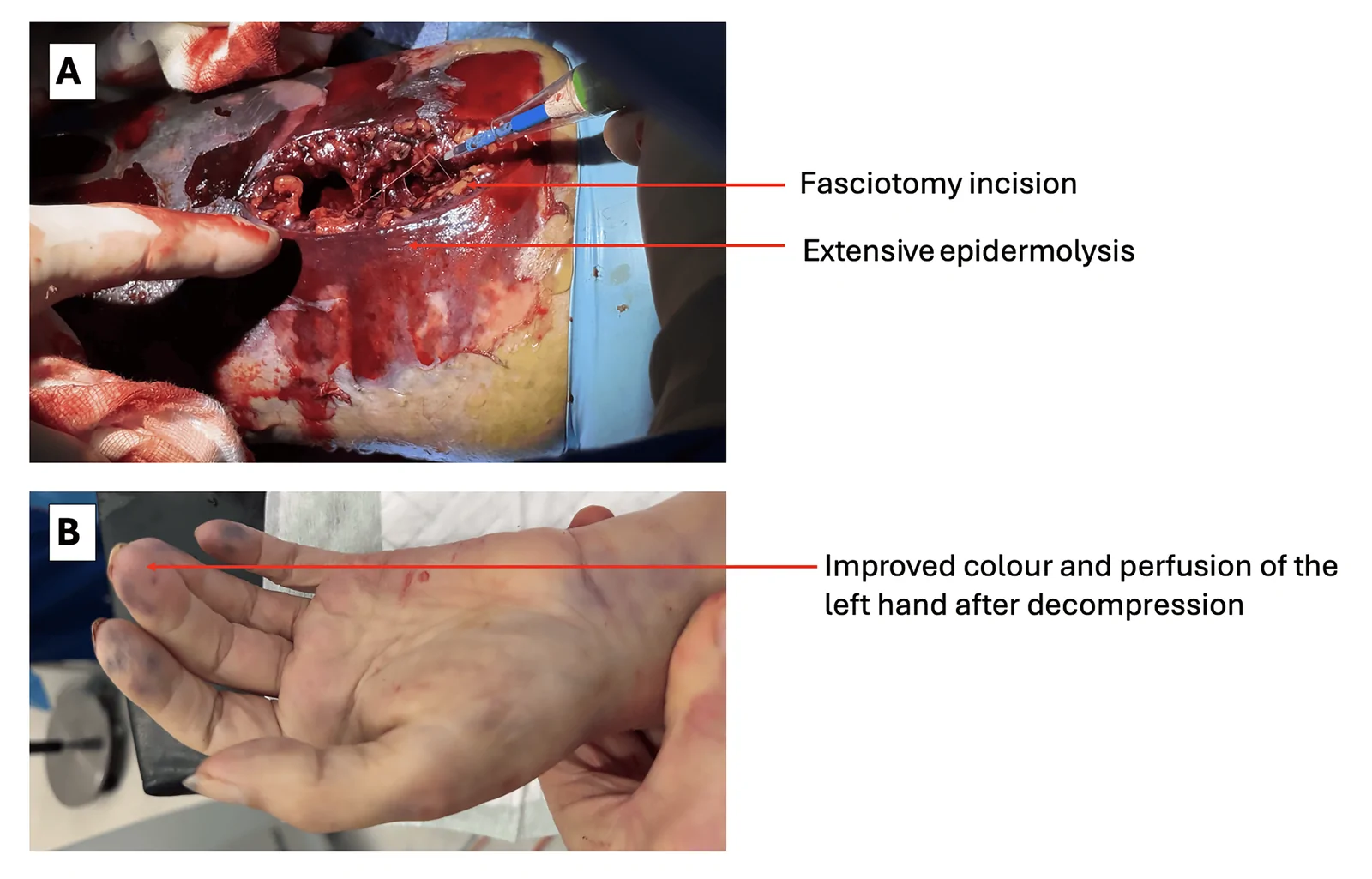
Surgical decompression and early improvement in distal perfusion. (A) Longitudinal decompression incision over the distal anterior upper arm with surrounding epidermolysis, haemorrhagic staining, and oedematous tissue; approximately 50-100 mL of haematoma was evacuated. (B) Improved colour of the left hand approximately 30 minutes after decompression; the radial pulse had returned immediately after fascial release, and capillary refill, previously absent, had improved to approximately four seconds. The photographs are used with the patient's written informed consent.

Emergency surgical decompression was undertaken in parallel with the ongoing liver transplantation, commencing approximately 15 minutes after recognition of the limb injury. Following sterile preparation and draping of the affected upper limb, a longitudinal incision was made over the distal upper arm and extended across the antecubital fossa as clinically required. Skin and subcutaneous tissues were divided, and approximately 50-100 mL of organised and liquefied haematoma was evacuated from the subcutaneous plane and anterior compartment. The deep brachial fascia was identified and opened longitudinally to achieve full decompression of the anterior compartment. Both the biceps and brachialis muscles were inspected. The biceps was contused but remained viable on assessment of colour, consistency, contractility, and capillary bleeding. No muscle debridement was required. The incision was extended distally to allow direct assessment of the antecubital region and proximal volar forearm. Although the cutaneous changes extended into the proximal forearm, the forearm flexor compartment was soft on direct palpation and did not require fascial release. The posterior compartment of the upper arm, the extensor compartment and mobile wad of the forearm, and the intrinsic compartments of the hand were assessed clinically by the plastic surgical team and were soft and compressible; formal compartment-pressure measurement was not undertaken. Carpal tunnel release was not performed. Immediately after release of the deep brachial fascia, compartment tension decreased and the radial pulse, previously absent, became palpable and remained so thereafter. Hand colour improved progressively, and capillary refill, which had been absent, was approximately four seconds, 30 minutes after decompression (Figure 2). Sterile Doppler examination confirmed brachial and ulnar arterial flow. Haemostasis was secured, the wound was left open because of residual oedema, and a sterile negative-pressure wound therapy dressing was applied, with planned delayed primary closure after resolution of swelling. The transplant was then completed.

Total intraoperative resuscitation during transplantation was approximately 24 L, comprising eight units of packed red blood cells (approximately 2.1 L), six units of fresh frozen plasma (approximately 1.7 L), 10 units of cryoprecipitate (approximately 0.4 L), 4 L of crystalloid, 800 mL of 20% albumin, and approximately 15 L of processed autologous cell-salvaged blood. Because cryoprecipitate is not compatible with administration through the Belmont RI-2 rapid infuser, it was transfused separately through a dedicated peripheral intravenous line and did not pass through the Belmont circuit. Accordingly, the approximately 4.5 L delivered through the affected left RIC represented only a proportion of the patient's overall intraoperative resuscitation burden.

After transplantation, the patient returned to the intensive care unit, where the limb was monitored during the early postoperative period with hourly neurovascular observations (skin colour and temperature, capillary refill, palpation of the radial pulse, and assessment of compartment tension), continuous pulse oximetry from the left hand, and at least daily review by the plastic surgical team. The radial pulse remained palpable throughout, and by postoperative day three, the anterior compartment was soft and capillary refill was less than three seconds. Serial clinical and biochemical surveillance, including creatine kinase, potassium, and renal indices, was undertaken for reperfusion injury, rhabdomyolysis, and renal complications. The fasciotomy wound underwent delayed primary closure on postoperative day 4, after the oedema had subsided. At hospital discharge 29 days post transplant, the patient had full active range of motion at the elbow, wrist, and digits, with no motor or sensory deficit. At structured six-week follow-up in the plastic surgery clinic, the wound was well healed, and examination showed full, pain-free active range of motion of the elbow, forearm, wrist, and digits, grip strength symmetrical with the contralateral hand, intact light-touch sensation in the median, ulnar and radial nerve distributions, no intrinsic muscle wasting or flexion contracture, and a palpable radial pulse; the transient postoperative numbness and heaviness had resolved. Follow-up beyond six weeks is not yet available. The complete clinical timeline is summarised in Table 2.

**Table 2:** Clinical timeline. CRRT, continuous renal replacement therapy; ICU, intensive care unit; RIC, rapid infusion catheter.

Relative time	Clinical event	Key finding or action
~2 weeks before transplantation	Clinical deterioration while awaiting graft	Progressive encephalopathy, oliguric renal failure, and gastrointestinal bleeding; haemoglobin nadir 60 g/L.
Evening before transplantation	ICU admission	CRRT commenced; multidisciplinary decision to proceed with urgent transplantation.
Approximately 4 hours before surgery	First RIC attempt	8.5 Fr RIC attempted under ultrasound guidance at left antecubital fossa; guidewire resistance encountered and the attempt abandoned.
Immediately before incision	Second RIC insertion	8.5 Fr RIC placed at the same site under real-time ultrasound; intraluminal position confirmed and 20 mL saline flush was unremarkable.
Following commencement of pressurised infusion	High-volume resuscitation	Approximately 4.5 L delivered through the left RIC circuit over approximately 2 hours; the extravasated fraction could not be determined. No high-pressure alarm was documented during infusion through the affected circuit.
Approximately 2 hours after transfusion began	Unexpected physiological change	Potassium 6.1 mmol/L and lactate 5.2 mmol/L during dissection (radial arterial sample; identical values on immediate femoral re-sampling); retrospective review later showed progressive damping of the ipsilateral radial waveform.
Immediately thereafter	Limb injury recognised	Cool, dusky hand; absent radial pulse; tense anterior compartment of the upper arm; ecchymosis, epidermolysis and haemorrhagic bullae at the antecubital fossa and proximal forearm.
Approximately 15 minutes after recognition	Emergency decompression	Anterior compartment of the upper arm released; forearm flexor compartment and remaining compartments of the limb assessed clinically and not released; radial pulse returned immediately after fascial release.
Postoperative course	Recovery	Hourly neurovascular observations in the intensive care unit; anterior compartment soft and capillary refill <3 s by day 3; delayed primary closure on day 4; normal motor and sensory function at discharge (day 29) and at six-week follow-up.

## Discussion

This case describes severe RIC extravasation during liver transplantation that progressed to upper-limb ACS requiring emergency surgical decompression. Although the catheter appeared satisfactory at insertion, the injury developed after final positioning and draping, when the site was no longer visible. Insertion-time ultrasound and an unremarkable test flush confirmed catheter function only at that time; the absence of a pressure alarm did not establish continued intravascular delivery.

Possible mechanisms

The precise mechanism of extravasation cannot be proven. The earlier failed attempt may have caused local venous injury or created a false passage, thereby increasing susceptibility to later extravasation, but this remains a possible rather than an established cause; catheter displacement during positioning and draping, vessel injury at the second insertion, and coagulopathy may also have contributed. Guidewire resistance is not, however, specific for vessel-wall injury and may occur at a venous valve or because of needle, wire, or vessel geometry [5]. The appropriate response is therefore not to assume injury, but to stop, reassess needle and wire position with ultrasound and avoid force. When persistent or unexplained resistance occurs, and vessel trauma cannot be excluded, reuse of the same vein for subsequent pressurised high-flow infusion is difficult to justify when a safer alternative site is available.

Coagulopathy may also have contributed. In the 839-patient liver-transplant cohort reported by Porter et al., international normalised ratio (INR) was the only variable independently associated with RIC-related complications; an increase from 1.4 to 2.2 was associated with an adjusted odds ratio of 1.98 (95% CI: 1.10-3.56; p = 0.02) [8]. Although conventional coagulation tests do not fully represent haemostatic balance in cirrhosis [12], a markedly prolonged INR may still identify patients in whom local vascular injury is less well tolerated.

Delayed recognition

General anaesthesia and surgical draping remove many of the early clinical features on which ACS is usually recognised, and the problem is particularly relevant to liver transplantation, in which reliable upper-body high-flow access is often required for major transfusion. International liver-transplant recommendations specifically advise ultrasound-guided high-flow venous access with regular review of insertion sites for extravasation and haematoma [2]. Contemporary anaesthetic monitoring guidance likewise supports maintaining intravenous access sites visible whenever practical [13]. Where continuous visualisation is impossible, a planned and documented programme of reinspection after positioning changes and during high-volume infusion is a reasonable safety response to this predictable monitoring blind spot.

Previous reports support this concern. Pressurised crystalloid infusion through a peripheral catheter has caused upper-arm compartment syndrome requiring fasciotomy [11]. Intraoperative forearm compartment injury related to peripheral intravenous infiltration has also been described during cardiac surgery [14], and severe forearm and hand swelling after pressurised blood administration under general anaesthesia has been reported [15]. RIC-specific reports remain uncommon, but distal skin necrosis following an apparently uncomplicated 8.5 Fr RIC placement has been described [9]. Importantly, an Anesthesia Patient Safety Foundation report of RIC infiltration during liver transplantation documented smooth operation of the rapid-infusion system without high-pressure alarms despite flow rates of up to 400 mL/min [16]. This closely parallels the present case and demonstrates that apparently normal pump performance does not establish continued intravascular delivery.

Why the rapid infuser did not alarm

The absence of a high-pressure alarm is physiologically plausible and is an important safety observation. The Belmont RI-2 reduces the delivered flow rate as measured line pressure approaches the configured pressure limit. A high-pressure alarm is intended to identify excessive downstream resistance or obstruction; it is not designed to establish that the catheter remains intravascular [17]. If a catheter becomes extravascular while the catheter tip continues to communicate with a relatively compliant, low-resistance tissue plane, substantial fluid may leave the vascular space without generating the abrupt rise in circuit pressure required to trigger a high-pressure alarm. The upper arm provides a large soft-tissue reservoir in which infused blood and fluid can dissect through subcutaneous and fascial planes before tissue pressure becomes sufficiently high to impede flow. In the present case, this is the most plausible explanation for the absence of a pressure alarm despite progressive extravasation and subsequent compartment hypertension. This interpretation remains inferential because local tissue compliance and contemporaneous line-pressure values were not independently recorded.

Physiological indicators of evolving limb compromise

The biochemical abnormalities that prompted limb inspection were non-specific and had several competing explanations. Hyperkalaemia during liver transplantation is multifactorial and may arise from transfusion, renal dysfunction, acidosis, and graft reperfusion [18]. In this patient, oliguric renal failure with interruption of continuous renal replacement therapy and substantial red-cell transfusion could each have contributed to the rise in potassium, and systemic hypoperfusion and reduced hepatic lactate clearance could account for the rise in lactate; the event also occurred during dissection rather than at reperfusion. The identical values obtained from the femoral line excluded a local sampling artefact but did not identify the source. Whether regional ischaemia of the injured limb contributed cannot be determined, and we do not attribute these changes to limb ischaemia. Their clinical value was only that they were unexpected during the dissection phase and prompted examination of the concealed limb.

Progressive attenuation of the ipsilateral radial arterial waveform was another retrospective indicator of impaired distal perfusion. At our institution, a femoral arterial catheter is inserted for every liver-transplant recipient and is used for continuous pressure monitoring, because radial pressures may underestimate central arterial pressure during reperfusion and vasopressor therapy [19]; the radial catheter, which is readily accessible at the arm board, is used predominantly for blood sampling. Because the femoral trace remained stable and the radial catheter was not the primary pressure monitor, the change in the radial waveform was not acted upon. An unexpected deterioration in an arterial waveform distal to a concealed high-flow access site should prompt examination of the limb before being attributed to artefact or catheter dysfunction. Similarly, a pulse-oximetry probe placed on the hand distal to a concealed high-flow catheter provides continuous, if indirect, plethysmographic surveillance of distal perfusion, and loss or attenuation of that signal should prompt inspection of the limb. These observations are hypothesis-generating only; neither distal arterial waveform attenuation nor the plethysmographic signal has been validated as a screening test for extravasation or compartment hypertension.

Implications for clinical practice

**Figure 3:**
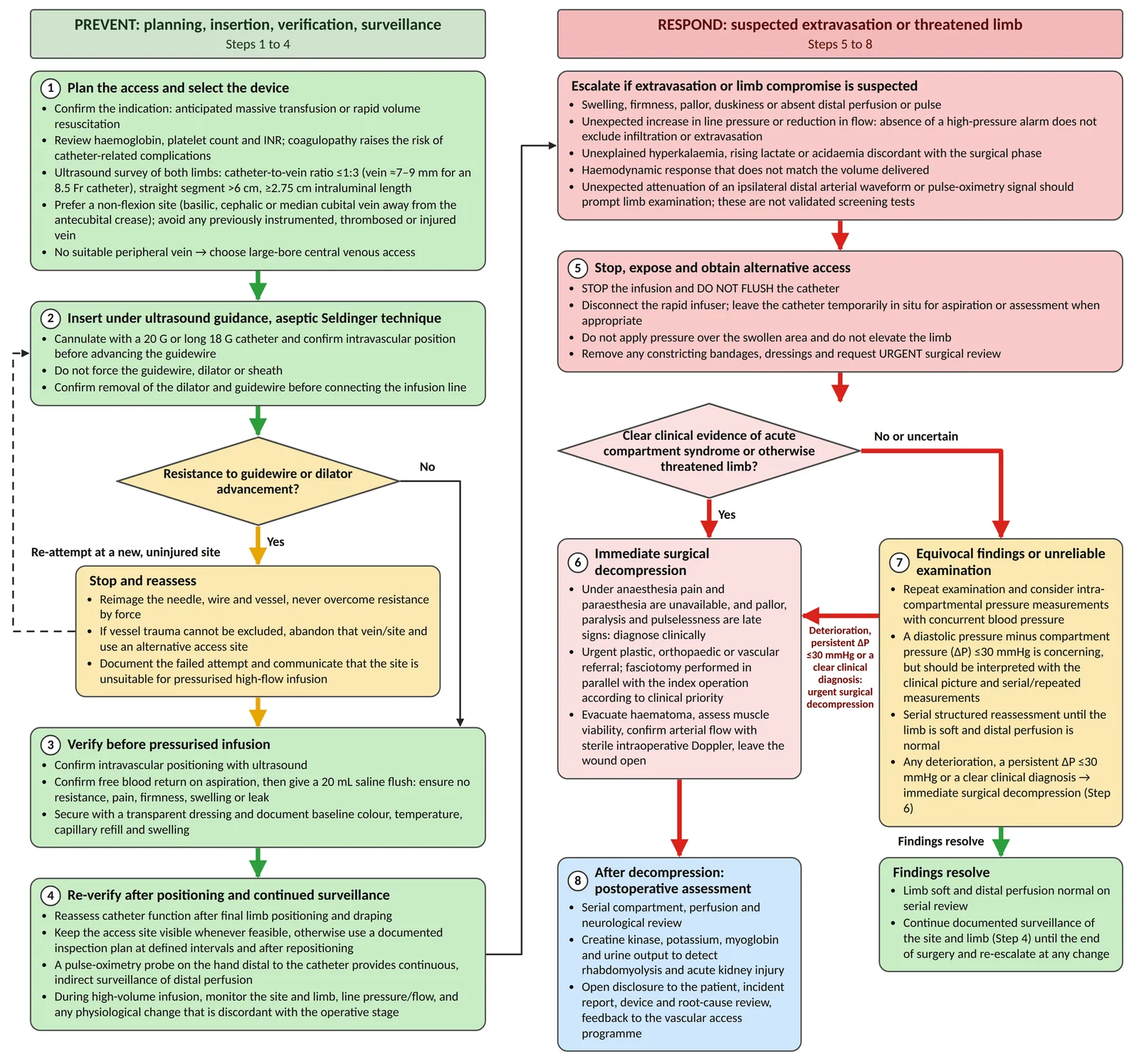
Proposed pathway for selection, insertion, surveillance, and escalation of peripheral rapid infusion catheters. The pathway synthesises contemporary liver-transplant vascular-access recommendations, RIC technical guidance, manufacturer pressure-control characteristics, anaesthetic monitoring guidance, published RIC safety observations, and acute compartment-syndrome recommendations [2,5,13,16,17,20]. A normal line-pressure display or absence of a high-pressure alarm does not exclude extravasation. Immediate surgical decompression (step 6) leads directly to post-decompression assessment (step 8); where findings are equivocal (step 7), any deterioration, a persistent ΔP of 30 mmHg or less, or a clear clinical diagnosis mandates immediate decompression, whereas resolution of findings is followed by continued documented surveillance. The pathway is intended for local institutional adaptation and has not been prospectively validated. Figure prepared using Microsoft PowerPoint (Microsoft 365, version 16.112.3; Microsoft Corporation, Redmond, WA, USA). Fr, French; G, gauge; INR, international normalised ratio; RIC, rapid infusion catheter; ΔP, diastolic arterial pressure minus measured intra-compartmental pressure.

The proposed pathway in Figure 3 separates prevention from response. Before pressurised infusion, clinicians should assess the intended vessel and prior instrumentation, use real-time ultrasound, avoid forcing a guidewire or dilator, and verify intravascular function with ultrasound, free aspiration of blood, and an observed flush. Catheter function should then be rechecked after final positioning and draping. During use, the access site should remain visible whenever feasible or be inspected according to a defined local plan, with additional inspection after repositioning or any unexplained change in line pressure, flow, limb perfusion, or systemic physiology; a pulse-oximetry probe on the hand distal to the catheter is a simple, continuous adjunct to this surveillance. A normal line-pressure display or absence of a high-pressure alarm should never be interpreted as evidence that infusion remains intravascular [16]. Contemporary RIC literature also supports attention to vessel calibre, straight venous course, and avoidance of flexion sites where feasible [5].

If extravasation is suspected, pressurised infusion should be stopped immediately, and the catheter should not be flushed. The limb should be exposed, constricting dressings removed, alternative high-flow access established, and urgent senior surgical assessment obtained. A clear clinical diagnosis of ACS or threatened limb perfusion warrants decompression without delaying treatment for pressure measurement. Where examination is equivocal or unreliable, contemporary guidance supports serial assessment and consideration of intra-compartmental pressure measurement with concurrent blood pressure; a diastolic pressure minus compartment pressure (ΔP) of 30 mmHg or less is concerning but should be interpreted in the clinical context and, where appropriate, with repeated or continuous measurements [6,20]. Any deterioration during this period of observation, a ΔP that remains 30 mmHg or less, or the emergence of a clear clinical diagnosis should prompt immediate surgical decompression rather than continued observation (Figure 3).

Limitations

This report has the limitations inherent to a single case. The mechanism of extravasation is inferred rather than proven, and the relative contributions of the earlier failed cannulation, subsequent catheter movement, local vessel injury, and coagulopathy cannot be separated. Although approximately 4.5 L was delivered through the affected circuit before recognition, the volume that actually extravasated is unknown. The biochemical abnormalities were non-specific, had several competing explanations in this patient, and may be absent in other cases. Compartment pressure was not measured; the diagnosis and decision to decompress were clinical because the limb already demonstrated marked tension, impaired distal perfusion, and established tissue injury. Follow-up was limited to six weeks; later sequelae such as fibrosis, contracture, or persistent neuropathic symptoms cannot be excluded, and our statements about recovery are confined to that period. Finally, the pathway in Figure 3 is a proposed synthesis of current guidance and the lessons of this case and has not been prospectively validated.

## Conclusions

Pressurised infusion through a large-bore peripheral catheter can produce a limb-threatening extravasation injury despite apparently satisfactory insertion-time verification and normal pressure-alarm behaviour. Persistent or unexplained guidewire resistance should prompt reassessment, and a vein in which vessel trauma cannot be excluded should not be reused for pressurised high-flow infusion when safer access is available. Ultrasound confirmation and an unremarkable test flush establish catheter position only at that moment; function should be reverified after final positioning, and high-flow peripheral sites should remain visible whenever feasible or undergo planned reinspection throughout use. Pressure alarms are important safeguards against excessive resistance and line occlusion, but their absence does not exclude extravasation into compliant soft tissues. Unexplained physiological deterioration should prompt examination of concealed access sites. In this patient, prompt intervention following recognition, with immediate cessation of infusion and early surgical decompression, was followed by full functional recovery at six-week follow-up.

## References

[1] Kashimutt S, Kotzé A (2017). Anaesthesia for liver transplantation. BJA Educ.

[2] Fernandez TM, Schofield N, Krenn CG (2022). What is the optimal anesthetic monitoring regarding immediate and short-term outcomes after liver transplantation?—A systematic review of the literature and expert panel recommendations. Clin Transplant.

[3] Wrenn EA, Wohlers R, Montgomery M, Cobb H, Condra J, Tharpe S, Patil N (2017). Comparison of flow dynamics of peripherally and centrally inserted intravenous catheters using a rapid infusion system (ThermaCor 1200). AANA J.

[4] Schumann R, Mandell MS, Mercaldo N (2013). Anesthesia for liver transplantation in United States academic centers: intraoperative practice. J Clin Anesth.

[5] Scorer A, Chahal R, Ellard L, Myles PS, Bradley WP (2025). Effective utilisation of rapid infusion catheters in perioperative care: a narrative review. BJA Open.

[6] von Keudell AG, Weaver MJ, Appleton PT (2015). Diagnosis and treatment of acute extremity compartment syndrome. Lancet.

[7] Kalyani BS, Fisher BE, Roberts CS, Giannoudis PV (2011). Compartment syndrome of the forearm: a systematic review. J Hand Surg Am.

[8] Porter SB, Hughes AJ, Ball CT, Hex KO, Brigham TJ, Pai SL (2021). Complications of peripherally inserted, large-bore, rapid-infusion catheters in orthotopic liver transplant patients. Transplant Proc.

[9] Chou WH, Rinderknecht TN, Mohabir PK, Phillips AW (2019). Skin necrosis distal to a rapid infusion catheter: understanding possible complications of large-bore vascular access devices. Cureus.

[10] Tobias MD, Hanson CW 3rd, Heppenstall RB, Aukburg SJ (1991). Compartment syndrome after pressurized infusion. Br J Anaesth.

[11] Willsey DB, Peterfreund RA (1997). Compartment syndrome of the upper arm after pressurized infiltration of intravenous fluid. J Clin Anesth.

[12] Tripodi A, Mannucci PM (2011). The coagulopathy of chronic liver disease. N Engl J Med.

[13] Klein AA, Meek T, Allcock E (2021). Recommendations for standards of monitoring during anaesthesia and recovery 2021: guideline from the Association of Anaesthetists. Anaesthesia.

[14] Garg LR, Chhabra S, Singla GK, Lakhwani S (2015). Acute compartment syndrome of the forearm in a patient undergoing coronary artery bypass surgery. Indian J Anaesth.

[15] Sung CY, Chung RK, Ra YS, Lee HS, Lee GY (2011). Impending compartment syndrome of the forearm and hand after a pressurized infusion in a patient under general anesthesia -a case report-. Korean J Anesthesiol.

[16] (2026). Anesthesia Patient Safety Foundation. Perils and pitfalls with the rapid infusion catheter (RIC). https://www.apsf.org/article/perils-and-pitfalls-with-the-rapid-infusion-catheter-ric/.

[17] Belmont Medical Technologies (2021). The Belmont® Rapid Infuser RI-2 Operator's Manual. The Belmont® Rapid Infuser RI-2 Operator's Manual.

[18] Chang W, Xu MR, George A (2025). Hyperkalemia in liver transplantation. J Clin Anesth.

[19] Arnal D, Garutti I, Perez-Peña J, Olmedilla L, Tzenkov IG (2026). Radial to femoral arterial blood pressure differences during liver transplantation. Anaesthesia.

[20] American Academy of Orthopaedic Surgeons (2026). American Academy of Orthopaedic Surgeons: Management of acute compartment syndrome: evidence-based clinical practice guideline.. Management of Acute Compartment Syndrome: Evidence-Based Clinical Practice Guideline.

